# “WE TREAT EVERYONE THE SAME”: PROVIDER PERSPECTIVES ON LGBT+ AGING IN EAST TENNESSEE

**DOI:** 10.1093/geroni/igae098.1200

**Published:** 2024-12-31

**Authors:** Joseph Winberry

**Affiliations:** University of North Carolina at Chapel Hill, Durham, North Carolina, United States

## Abstract

Aging Services providers in East Tennessee were asked to report barriers and solutions they saw to 5 aging-related needs raised by local Lesbian, Gay, Bisexual, and Transgender plus (LGBT+) people fifty years and older. The 5 needs included services for caregivers, social support, culturally competent care, LGBT+ Specific Services, and Planning. Fourteen providers working in settings such as offices on aging and long-term care facilities shared 248 responses via a qualitative survey. Thematic analysis of those responses revealed 6 barrier themes (ageism and elder abuse, discrimination, fewer personal resources, identity concealment, staff limitations, and stigma) and 4 solution themes (direct affirmation, creating and sharing information in partnership, inclusive written messages and images, and training participation). Review of the data also indicated a third category of response which was denial of needs raised. The findings indicate that providers have a varied understanding of barriers and potential solutions for meeting the aging services needs of LGBT+ older adults. They also demonstrate a lack of awareness among at least some providers of how LGBT+ older adults feel about their ability to access aging services in comparison to non-LGBT+ older adults. These findings are part of a larger, multidisciplinary community-engaged participatory research project that is ongoing in East Tennessee. Its purpose is to address information and social barriers that LGBT+ older adults see as obstructing their ability to age with dignity and respect in the community they call home. The findings raised from provider survey responses are useful for designing future interventions for providers.

